# One Surface Treatment, Multiple Possibilities: Broadening the Use-Potential of Para-Aramid Fibers with Mechanical Adhesion

**DOI:** 10.3390/polym13183114

**Published:** 2021-09-15

**Authors:** Sarianna Palola, Farzin Javanshour, Shadi Kolahgar Azari, Vasileios Koutsos, Essi Sarlin

**Affiliations:** 1 Materials Science and Environmental Engineering Unit, Faculty of Engineering and Natural Sciences, Tampere University, FI-33014 Tampere, Finland; farzin.javanshour@tuni.fi (F.J.); essi.sarlin@tuni.fi (E.S.); 2 School of Engineering, Institute for Materials and Processes, The University of Edinburgh, The King’s Buildings, Robert Stevenson Road, Edinburgh EH9 3FB, UK; s.kolahgarazari@ed.ac.uk (S.K.A.); vasileios.koutsos@ed.ac.uk (V.K.)

**Keywords:** aramid fibers, surface modification, adhesion, interphase, interfacial shear strength

## Abstract

Aramid fibers are high-strength and high-modulus technical fibers used in protective clothing, such as bulletproof vests and helmets, as well as in industrial applications, such as tires and brake pads. However, their full potential is not currently utilized due to adhesion problems to matrix materials. In this paper, we study how the introduction of mechanical adhesion between aramid fibers and matrix material the affects adhesion properties of the fiber in both thermoplastic and thermoset matrix. A microwave-induced surface modification method is used to create nanostructures to the fiber surface and a high throughput microbond method is used to determine changes in interfacial shear strength with an epoxy (EP) and a polypropylene (PP) matrix. Additionally, Fourier transform infrared spectroscopy, atomic force microscopy, and scanning electron microscopy were used to evaluate the surface morphology of the fibers and differences in failure mechanism at the fiber-matrix interface. We were able to increase interfacial shear strength (IFSS) by 82 and 358%, in EP and PP matrix, respectively, due to increased surface roughness and mechanical adhesion. Also, aging studies were conducted to confirm that no changes in the adhesion properties would occur over time.

## 1. Introduction

Para-aramid, poly (*p*-phenylene terephthalamide), fibers are highly crystalline synthetic fibers with high tensile strength, excellent chemical and abrasion resistance and high melting point. They even outrank carbon fiber in impact and wear resistance while also having higher a strength-to-weight ratio [1,2,3]. Aramid is used in fibrous form as well as in woven textile and pulp form, as reinforcement in demanding composite applications ranging from protective clothing (helmets, bulletproof vests, and fire protection) to automotive and industrial applications (gaskets, brake pads, tires, conveyor belts, and hoses).

However, the full use-potential of aramid fiber is hindered due to adhesion issues to matrix materials. To achieve the high strength-to-weight ratio, outstanding mechanical performance and durability characteristic of advanced composite materials [1,2,3], strong adhesion between the reinforcing fibers and the matrix material is critical. The adhesion issues with aramid fibers arise from the surface structure of the fiber, which is very smooth and chemically inert, lacking in reactive side groups [4,5]. To overcome this phenomenon, surface treatments are used, which traditionally promote either physical or chemical adhesion with the matrix. For example, a plasma treatment increases the surface energy of the fiber by increasing hydrogen bonds at the fiber surface, thus enabling a physical bond to be formed between the fiber and matrix [6,7,8]. On the other hand, with a chemical surface treatment, reactive side groups are grafted to the fiber surface, which can react with the matrix material and create a strong covalent bond between the fiber and the matrix [9,10]. However, these methods are often suitable for only one type of matrix material [11,12,13], may lose their effectiveness rapidly during storage [7] and may drastically reduce the mechanical properties of the fibers [14,15,16]. Thus, new approaches are needed, and research is increasingly directed towards utilizing mechanical adhesion between fibers and matrix [17].

Typically, mechanical adhesion is considered a lesser form of adhesion in composites, but it has some major advantages, such as independence of chemical compatibility. With mechanical adhesion or interlocking as the prominent adhesion mechanism, a wider range of material combinations could be used in composite applications, including thermoplastics. Thermoplastic materials are a desirable group of matrix materials for composite applications due to their lower toxicity and easier recyclability when compared to thermosetting materials. However, they are a challenging material group in terms of adhesion. Another benefit of mechanical adhesion at the fiber-matrix interface is that composite production becomes more economical as the same surface treatment can be used with multiple matrix types.

Mechanical adhesion or interlocking can be formed between the fiber and matrix, for example, by adding nanowires [18,19,20], nanoparticles [21,22,23], nanotubes [13,24,25,26], or nanofibers [27,28,29] to the fiber surface. These structures simultaneously increase the surface area and the surface roughness of the fiber. For example, by increasing mechanical adhesion together with chemical interactions, Nasser et al. [30] have been able to increase short beam strength of laser-induced graphene-coated aramid fabric by 70% in epoxy matrix. Lv et al. [13] have achieved similar results with in-situ polymer grafting and carbon nanotubes on aramid in the epoxy matrix, but they concluded the increase in interfacial shear strength (IFSS) to be due to increased polarity rather than topography. However, by purely increasing mechanical adhesion with adsorbed aramid nanofibers, Nasser et al. [27] have been able to increase short beam strength by 26% and IFSS by 70% in epoxy, which shows what the imminent potential mechanical adhesion has in terms of composite applications.

However, to fully benefit from mechanical adhesion, the attached medium (i.e., nanofibers or particles) needs also to be strongly adhered to the fiber surface, as Gonzalez-Chi et al. [24] and Ehlert et al. [20] have demonstrated. Also, the unique skin-core structure of the highly crystalline para-aramid fiber may lower the overall adhesion properties of the fiber even if strong interphase is formed between the fiber and matrix [31]. As force is applied to the interphase, the top layer of the fiber may fibrillate and be sheared off completely. By applying a “new skin” layer of graphene to the fiber, Cheng et al. [32] have been able to reconfigure the phenomenon and change the failure mechanism from fibrillation of the fiber “skin” to clean fracture at the interface, while increasing the IFSS by 75% in epoxy.

In this paper, we study the effect of mechanical adhesion as the main adhesion mechanism at the fiber-matrix interface. This is done by adding nanoscale deposits onto aramid fiber surface that increase surface area and topography and thus, enable mechanical adhesion at the fiber-matrix interphase. The concept of nanoscale deposit addition to increase adhesion in macroscale has been proven effective in our previous study [33]. However, the question remained whether the increased adhesion was purely due to mechanical adhesion or a combined effect (i.e., secondary entanglement) and would the result really be effective with other matrix materials as well. In this paper, we aim to address these questions and show that the effect is universal and does work with multiple matrix material types, and that the adhesion increase is purely due to increased mechanical adhesion. Also, we show that the effect is similar across different length scales ranging from micro to macroscale. Both thermoplastic and thermoset matrices were used to evaluate reliably the behavior of the nanodeposit decorated fiber surface in different matrix types, which have significantly different chemical and physical properties. Micromechanical testing is applied so that the failure mode and mechanism of the interphase can be monitored more closely and the effect of secondary artefacts, which may be present in macroscopic bulk material testing, such as fiber entanglement, can be eliminated from the results. For this, a high throughput microbond test system [34] was used to measure the IFSS of these nano-deposit decorated fibers. This test method was chosen over the more traditional fiber fragmentation test because fiber fragmentation test is unsuitable for aramid fibers due to their high-strain tensile failure mode [31]. Also, the microbond test method can be applied more easily to both thermoplastic and thermoset matrix materials. In order to focus on the effect of mechanical adhesion, polypropylene (PP) was chosen as the thermoplastic matrix material. PP has very limited hydrogen bonding interactions with the fiber surface, thus making it ideal for this type of investigation. Epoxy (EP) was used as the thermoset matrix because of its availability and wide use in polymer composites across the field. Fourier transform infrared spectroscopy (FTIR), scanning electron microscopy (SEM), and atomic force microscopy (AFM) were used to characterize the nanostructures, study the fiber-matrix interphase and identify the failure mechanism. Further, it was also investigated how well the widely debated microbond methodology represents macroscale properties of the composite by comparison to the previous results [33]. Also, the influence of aging during storage is studied, and what effect it has on the effectiveness of the surface treatment.

## 2. Materials and Methods

### 2.1. Aramid Surface Modification

Para-aramid fibers, Twaron 2201 (Teijin, Amsterdam, The Netherlands; properties according to the supplier: tensile strength 2.1 N/tex, linear density 1610 dtex, ~0.15 w-% sizing), were used as the fiber material. Prior to the surface treatment, the fibers were washed with mild detergent and rinsed with ethanol to remove the water-soluble, EO and PO alcohol component containing, surface sizing, and finally dried thoroughly. These fibers are denoted with suffix W, as ‘washed’. Microwave-induced surface treatment was applied to a section of the washed fibers. This was done by first carburizing the fibers with Agar Turbo carbon evaporator B7230 (Agar Scientific, Stansted, UK) and then placing them into a glass container together with reactive chemicals (1:1 graphite and ferrocene). The container is then sealed and placed into a microwave oven, as described in the previous study [33]. Irradiation time of 14 s is used as it has been [33] determined to yield the best distribution and coverage of the fiber surface with the nanodeposits. These fibers are denoted with suffix MW. To investigate the storage properties of the MW—fibers, some of the fibers were kept in air at room temperature, protected from light, for 48 months prior to testing. Sample nomenclature is presented in Table 1.

### 2.2. Interfacial Shear Strength

IFSS was measured with the microbond test method [35], in which single fiber microcomposite samples are prepared and tested. The samples are prepared by depositing droplets of the matrix material onto single fiber filaments and allowed to cure or cool down depending on the material type used. The droplets are then individually loaded with microvise blades until the droplet is detached from the fiber. To calculate the IFSS, the load required to detach the droplet (*F_max_*) is then compared with the area of the fiber surface embedded by the droplet (*A_emb_*), as described by Equation (1).

(1) $$\frac{F_{max}}{A_{emb}}=\mathrm{IFSS}$$

For the IFSS testing in thermoset matrix, a low-viscosity epoxy (EP) resin system Araldite LY 5052/Aradur 5052 (Huntsman, Ratingen, Germany) was used, with a mixing ratio of 100/38, respectively. The resin was cured for 24 h at room temperature, followed by post-curing at 60 °C for 12 h. For the IFSS testing in thermoplastic matrix, a high melt flow heterophasic copolymer, polypropylene (PP) BJ380MO (Borealis AG, Vienna, Austria), was used.

Epoxy and PP droplets were dispensed onto the fibers with FIBROdrop (Fibrobotics Oy, Tampere, Finland) setup. A computer-controlled aluminum heating element was used to achieve optimum melt flow during PP droplet deposition. To prevent oxidation and thermal degradation of the PP melt during the droplet deposition, the FIBROdrop device was placed into an air-tight cabinet filled with nitrogen (N_2_) gas. Also, a new batch of polymer melt was prepared for each fiber. High-throughput FIBRObond (Fibrobotics Oy, Tampere, Finland) [34] device was used for microbond measurements with a 1 N S-beam load cell and stainless steel sample holder. Testing was done in air at room temperature. Five fibers per sample type with 20–40 droplets per fiber were tested, resulting in approximately 100–200 data points for each sample type.

### 2.3. Microscopy

Field emission gun SEM Zeiss ULTRAplus (Zeiss, Oberkochen, Germany) was used for detailed imaging of the fiber surface and of the failed interface after IFSS testing. To minimize charging and to improve image quality, the samples were attached to aluminum sample holders with carbon glue and coated with carbon and tiny amount of gold.

Surface topography of the fibers was studied with an AFM MultiMode Nanoscope IIIa (Bruker, Santa Barbara, CA, USA). The measurements were done with tapping mode to gain information about possible phase shifts together with high spatial resolution while limiting the effect of artifacts and sample damage. Antimony (n) doped silicon tips of 0.01–0.025 Ohm-cm (model: NTESPA, Bruker, Santa Barbara, CA, USA) were used, which had a reflective aluminum coating on the backside. The imaging was done in air at room temperature. For the imaging process, samples were attached to a magnetic disc with double-sided adhesive. Data was analyzed with Gwyddion software [36].

### 2.4. FTIR Spectroscopy

The aramid fiber surface was analyzed with FTIR spectroscope Spectrum One (Perkin-Elmer, Buckinghamshire, UK). The Universal Attenuated Total Reflectance (ATR) sampling accessory of FTIR had a Diamond/ZeSe crystal with a 1.66 µm depth of penetration. Transmittance spectra were recorded within the 4000 to 600 cm^−1^ range and a 0.5 cm^−1^ resolution.

## 3. Results and Discussion

**Figure 1:**
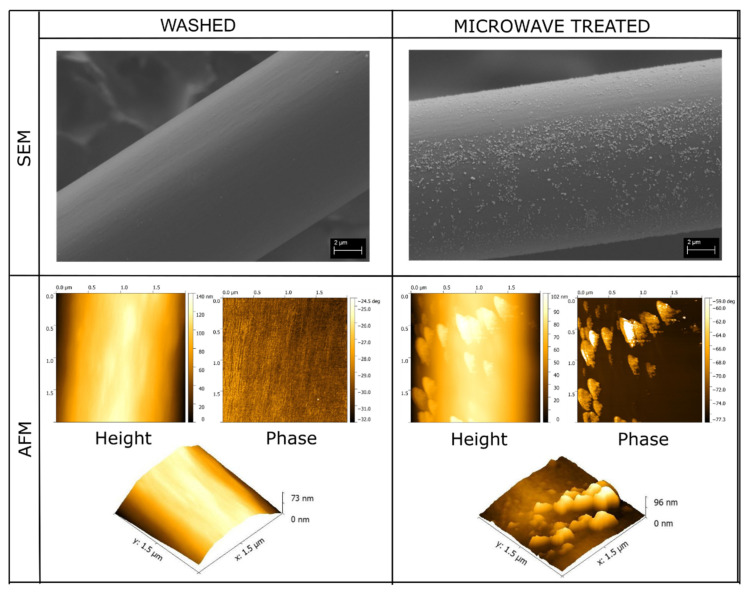
SEM images and corresponding AFM images of the aramid fibers before and after the microwave surface treatment.

The fiber surface after the microwave irradiation treatment revealed an abundance of nanostructures covering the surface. As seen in Figure 1, the nanostructures are of irregular shape and that the topography of the fibers has changed due to the surface treatment significantly, but no visible voids are generated on the fiber surface. This is in line with our previous findings stating that the treatment has no negative effect on the mechanical properties of the fibers [33] and highlights the repeatability of the surface treatment method.

**Figure 2:**
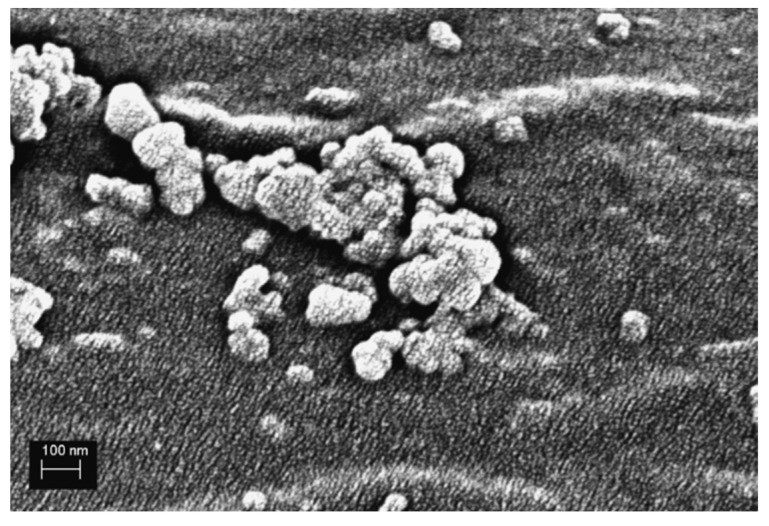
SEM EsB image of the microwave treated fiber surface highlighting the Z-contrast between the fiber surface and nanostructures.

The AFM studies supported the SEM findings depicting clearly defined protrusions on the fiber surface. The phase contrast image highlights the structural and chemical difference between the bulk fiber and the nanostructures. As the color gradient in AFM phase contrast image is a combination of topographical details as well as changes in mechanical and adhesive properties, a contrast in color is created when the chemical and physical properties change in the imaged area. As the nanostructures appear brighter than the fiber surface, it can be deduced that they are not the same material as the fiber surface. Additionally, when using an Energy selective Backscattered (EsB) detector with SEM, the nanostructures also appear lighter than the bulk fiber itself, as seen in Figure 2. The EsB detector reduces edge contrast in the image and thus, the apparent color difference between the bulk fiber and the nanostructures is due to increased Z-contrast [37] between the two. This, together with the AFM findings, means that the nanostructures are of different material and added to the surface during the microwave surface treatment rather than coming from the bulk fiber itself due to wrinkling or surface degradation.

The increased Z-contrast also implies that the nanostructures are mostly carbon-based compounds with traces of iron from ferrocene used in the microwave treatment. As a heavier element, iron would show up lighter in the EsB image, as seen in Figure 2. The iron molecule in ferrocene acts as a nucleation site for the carbon atoms as it is heated up during the treatment process [38] and thus can accumulate into the nanostructures. As seen from the figures, the irregular shape and varying size of the nanostructures increases the surface area of the fiber efficiently. This increases frictional forces at the fiber-matrix interface as well as adhesion through mechanical interlocking.

**Figure 3:**
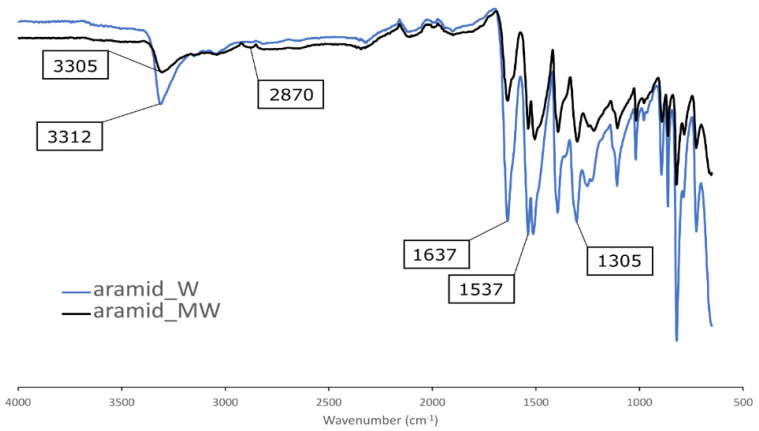
FTIR transmittance spectrum of washed (W) and microwave surface-treated (MW) aramid fibers.

The FTIR spectrum (Figure 3) of the washed aramid fibers reveals characteristic peaks for para-aramid at 3312 cm^−1^ (–NH, hydrogen bond association states), 1637 cm^−1^ (C=O stretching vibration band of amide), 1537 cm^−1^ (N–H curved vibration), and 1305 cm^−1^ (N–H bending vibration) [39,40,41]. Compared to the FTIR spectrum of W-fibers, the hydrogen band peak of MW-fibers has broadened and moved to a lower wavenumber of 3305 cm^−1^ indicating increased hydrogen bonding at the surface and weakened hydrogen bonding in the polymer chains of the aramid fiber skin layer [40]. This means that the intense heat during the surface treatment causes some damage to the fiber surface but not to a degree that would affect the tensile properties of the fibers, as shown previously [33], or be visible in SEM. Also, a new peak is present at 2870 cm^−1^, indicating CH_2_/CH groups at the fiber surface [40]. The same peak is also present in ferrocene and graphite [42,43]. This confirms that the nanostructures are decomposition products of ferrocene and graphite, formed during the microwave irradiation treatment.

**Figure 4:**
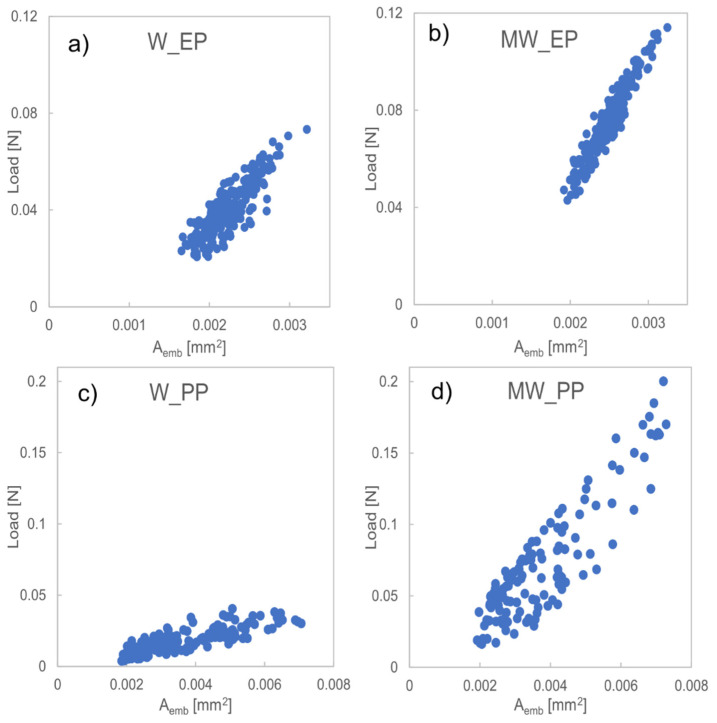
Load vs. embedded area for all measured droplets for (**a**) W_EP, (**b**) MW_EP, (**c**) W_PP, and (**d**) MW_PP.

IFSS was calculated with linear regression using the slope of load versus embedded area (Aemb) for each tested fiber separately. The IFSS for each sample type was then taken by calculating the average of the IFSS values of the separate fibers of that sample type. From the data in Figure 4, it can be seen that the load required to debond a droplet is higher with samples that are covered with nanostructures than with those that are not, even though the effective embedded area is similar. This implies that protrusions as small as nanoscale, can significantly alter the properties of the fiber-matrix interface in a way that can be detected with a microscale method. This same trend can be seen with both EP and PP matrix. As the behavior is similar in both thermoset and thermoplastic matrix, it emphasizes the importance of mechanical adhesion as a major adhesion mechanism that is independent of chemical compatibility. By increasing mechanical adhesion with the nanostructures, the maximum load increased by 56 and 395% in MW_EP and MW_PP, respectively. Although, the scattering of data appears to increase due to the surface treatment in MW_PP as compared to W_PP, this is not the case. Relative standard deviation (RSD) in both data sets is similar (~14%), which means that the data is highly comparable. Also, the R2 value for all measured samples ranged between 0.82–0.98, meaning high compatibility with the linear fit and thus, highly reliable measurement results.

**Figure 5:**
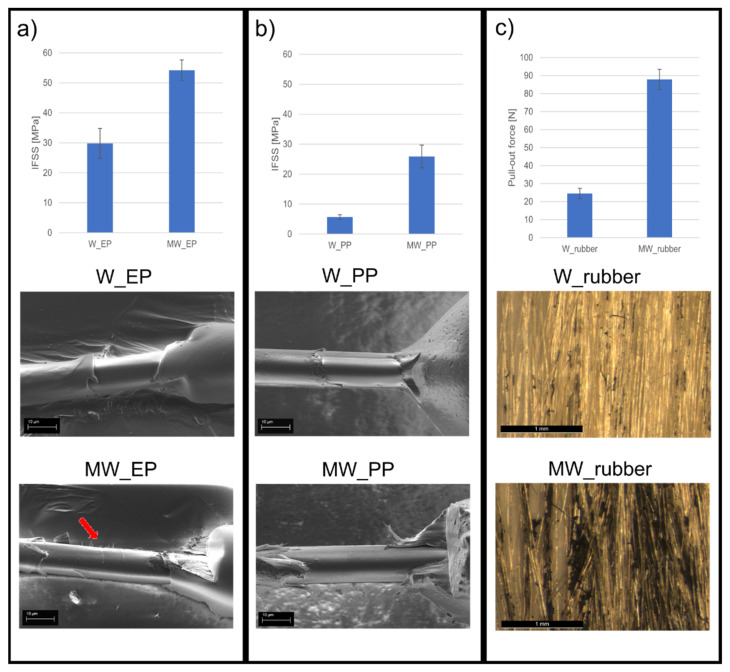
The IFSS results and SEM images of the corresponding fracture surfaces for (**a**) W_EP and MW_EP, where the arrow indicates fibrillation of the fiber surface, and (**b**) W_PP and MW_PP. The fiber bundle pull-out force and corresponding optical images are presented in (**c**) for W_rubber and MW_rubber [33].

The average IFSS results are presented in Figure 5 for W_EP, MW_EP, W_PP, and MW_PP together with macroscopic fiber bundle pull-out test results for the same surface treatment in rubber [33]. It is evident that the IFSS increases alongside with the increased surface topography of the fibers. Moreover, the increase in IFSS follows a similar trend with the bundle pull-out test in rubber. The results show that the IFSS increases in a similar fashion with thermoplastic, thermoset, and elastomeric matrices, even though the potential for chemical interaction of these matrix types is very different. For example, with EP, an increase in interfacial adhesion can be achieved through covalent bonding with the fiber surface during curing or by creating higher frictional force with the cured and cross-linked resin [13]. Mercaptan compounds, Lewis acid, and alkali products can be used to achieve such covalent bonds with EP. However, as none of them are grafted to the fiber surface in this case, what remains, is the increase in friction. This is also the case with PP. The chemical composition of PP provides only limited hydrogen interaction, which could affect favorably to interfacial adhesion with aramid. The main attribute towards the adhesion is mostly compressive forces due to favorable transcrystallization [31,39] occurring during the cooling process of the polymer melt. This was noted by Wang et al. [44]. They showed that small grooves and protrusions will increase thermal stress due to increased stress concentration during the PP crystallization when the melt is cooling. This localized stress concentration will further on enhance the nucleation ability of PP and, thus, promote transcrystallization leading to enhanced interfacial adhesion. The nanostructures created to the aramid fiber surface in this study, will act as such protrusions as described by Wang et al., and thus, lead to increased mechanical adhesion between the fiber surface and PP. In both EP and PP matrix, the nanostructures also increase stress transferability, which in turn, increases the IFSS in a similar fashion in both matrix types. These findings indicate that the primary adhesion mechanism between the fiber and polymer in this case, is indeed mechanical adhesion.

The measured IFSS value for W_PP is 5.7 MPa, which is similar to other studies [24] done with microdroplet test and aramid/PP combination. This shows that the high-throughput microbond method is highly suitable for IFSS evaluation also with thermoplastic matrix. Overall, the IFSS increased from 29.8 to 54.2 MPa (82% increase) and from 5.7 to 25.9 MPa (358% increase), in EP and PP matrix, respectively, due to the surface treatment. This is very significant as it shows that the surface treatment is suitable for both thermoplastic and thermoset materials and that it has a similar effect in them both. Also, it is worthwhile noting that with this straightforward and fast surface treatment process, the IFSS of aramid/EP combination could be brought to the same level as with other more complicated methods reported only recently [32].

**Figure 6:**
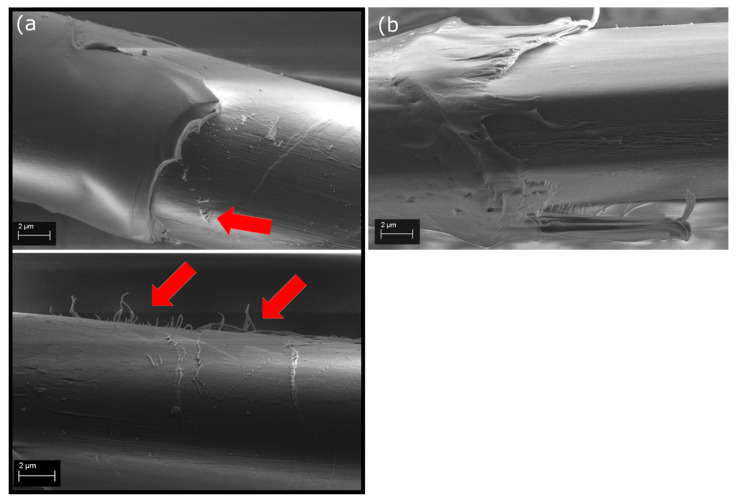
SEM images of the facture surfaces of (**a**) MW_EP, where red arrows indicate the fibrillation and peeling of the fiber surface, and (**b**) MW_PP after IFSS testing.

SEM images of the failed fiber-matrix interphase and visual observation during microbond testing supported the IFSS results. The failure mechanism during testing changed from pure shear at the interphase to a combination of peeling and shear as the surface topography was introduced (see videos in Supplementary Materials: Supporting Information files S1 and S2). With no surface treatment, the fiber surface after debonding appears smooth and unscathed, with only a minor amount of matrix residue remaining, as seen in Figure 5. This indicates that the fiber-matrix interphase has failed as the matrix droplet is sheared off. Also, the detachment site of the droplet shows a clean break with a small gap between the fiber surface and matrix, indicating a weak interphase. With the surface-treated fibers, the detachment site of the droplet shows no gap and is more uneven, as seen in Figure 6a,b, meaning that a stronger fiber-matrix interphase has been created. The debonded surface is rougher even with some fibrillation of the fiber skin structure, which indicates that the failure has shifted from purely occurring at the fiber-matrix interphase to a combination of fiber surface fibrillation and peeling together with interfacial shearing. The change in the appearance of the debonded surface is very clear with the harder EP matrix, as seen in Figure 5a and Figure 6a, where red arrows point to sections of fibrillated fiber surface. Whereas with the softer PP, the matrix is rather fibrillating itself and clinging to the nanostructures than cleaving bits off from the fiber surface. The PP strands clinging to the fiber can be seen clearly underneath the fiber in Figure 6b. This indicates that the fiber/matrix adhesion is higher than the cohesive strength of the matrix. It also means, that the nanostructures are strongly attached to the fiber surface, as delamination occurs jointly from the skin-core interphase and skin-matrix interphase. As a result, it can be said that the main adhesion mechanism contributing towards the increased interfacial adhesion is mechanical adhesion.

Additionally, the IFSS results follow very closely the same trend observed with the macroscopic fiber bundle pull-out test in rubber [33]. By increasing the amount of nanodeposits on the fiber surface, the adhesion and the strength of the interphase can be increased in both micro and macroscale. This is in line with findings of previous studies, such as the ones made by Beter et al. [45]. It also suggests that the high-throughput microbond method produces reliable data, which can indeed be used to evaluate adhesion properties in macroscopic composite structures. This type of composite research and development process can be made more economical and efficient.

Storage properties, and more precisely, aging, of the nanostructure covered fiber surface was also investigated. Some of the surface-treated fibers were taken aside and kept for 48 months at room temperature and protected from light. The IFSS of these fibers was measured with the microbond procedure in EP, and visual changes in the fiber surface were studied with SEM. The results revealed only minimal decrease in IFSS (~2%), which is well within the deviation range, compared to newly surface-treated fibers. Also, no change in the appearance of the fiber surface was observed. Thus, no significant decrease in the interfacial properties of the fibers has occurred, and the surface treatment can be considered durable enough to withstand storage over long periods of time.

## 4. Conclusions

This work explored the effect of nanostructures on the interfacial adhesion of aramid fiber in both a thermoplastic and a thermoset matrix and related the results also to an elastomeric matrix from a previous study. Our findings demonstrated that a significant increase in IFSS can be achieved in both thermoplastic (+358%) and thermoset (+82%) matrix, while maintaining mechanical and storage properties of the fibers. The increase in IFSS was noted to be due to enhanced mechanical adhesion between the fiber surface and matrix material caused by the addition of nanostructures to the fiber surface. The positive effect of the nanostructures on interfacial strength was observed both in micro and macroscale tests. The failure mechanism of the fiber-matrix interphase changes from clean shear to combined shear and peeling, as the level of mechanical adhesion increases, proving that the nanostructures are strongly attached to the fiber surface. These results highlight the significance of mechanical adhesion as the main adhesion mechanism and expand the use-potential of aramid fibers to multiple matrix material types and applications with just one fiber surface treatment.

## Figures and Tables

**Table 1:** Sample nomenclature used in the study.

Sample Name	Matrix Material	Washing	Microwave Treatment
EP_W	epoxy	YES	NO
EP_MW	epoxy	YES	YES
PP_W	polypropylene	YES	NO
PP_MW	polypropylene	YES	YES

## Data Availability

The data presented in this study are available on request from the corresponding author.

## Supplementary Materials

The following are available online at https://www.mdpi.com/article/10.3390/polym13183114/s1, Supporting Information file S1: A video of debonding of PP droplet on W_PP. Supporting Information file S2: A video of debonding of PP droplet on MW_PP.
